# Cranberry Juice Extract Rapidly Protects Demineralized Dentin against Digestion and Inhibits Its Gelatinolytic Activity

**DOI:** 10.3390/ma14133637

**Published:** 2021-06-29

**Authors:** Yong Wang, Austin Green, Xiaomei Yao, Hang Liu, Saleha Nisar, Jeffrey Paul Gorski, Viviane Hass

**Affiliations:** Department of Oral & Craniofacial Sciences and Center of Excellence in Mineralized Tissues, School of Dentistry, University of Missouri-Kansas City, 650 E 25th St., Kansas City, MO 64108, USA; ajgc95@mail.umkc.edu (A.G.); yaoxm2000@gmail.com (X.Y.); hangliu1984@gmail.com (H.L.); haqs@umkc.edu (S.N.); GorskiJ@umkc.edu (J.P.G.); Hassv@umkc.edu (V.H.)

**Keywords:** dentin, collagen cross-linking, cranberry juice extract, green tea, carbodiimide, grape seed extract, polyphenols, collagenase, MMPs

## Abstract

Improving the longevity of composite restorations has proven to be difficult when they are bonded to dentin. Dentin demineralization leaves collagen fibrils susceptible to enzymatic digestion, which causes breakdown of the resin–dentin interface. Therefore, measures for counteracting the enzymatic environment by enhancing dentin collagen’s resistance to degradation have the potential to improve the durability of dental composite restorations. This study aimed to evaluate the effects of polyphenol-rich extracts and a chemical cross-linker on the cross-linking interaction, resistance to digestion, and endogenous matrix metalloproteinase (MMP) activities of dentin collagen under clinically relevant conditions. Ten-µm-thick films were cut from dentin slabs of non-carious extracted human third molars. Following demineralization, polyphenol-rich extracts—including grape seed (GSE), green tea (GTE), and cranberry juice (CJE)—or chemical cross-linker carbodiimide with n-hydroxysuccinimide (EDC/NHS) were applied to the demineralized dentin surfaces for 30 s. The collagen cross-linking, bio-stabilization, and gelatinolytic activities of MMPs 2 and 9 were studied by using Fourier-transform infrared spectroscopy, weight loss, hydroxyproline release, scanning/transmission electron microscopy, and in situ zymography. All treatments significantly increased resistance to collagenase degradation and reduced the gelatinolytic MMP activity of dentin collagen compared to the untreated control. The CJE- and GSE-treated groups were more resistant to digestion than the GTE- or EDC/NHS-treated ones (*p* < 0.05), which was consistent with the cross-linking interaction found with FTIR and the in situ performance on the acid-etched dentin surface found with SEM/TEM. The collagen films treated with CJE showed the lowest MMP activity, followed by GSE, GTE, and, finally, EDC/NHS. The CJE-treated dentin collagen rapidly increased its resistance to digestion and MMP inhibition. An application of CJE as short as 30 s may be a clinically feasible approach to improving the longevity of dentin bonding in composite restorations.

## 1. Introduction

Despite significant improvements in contemporary adhesive systems for the short term, the limited durability and stability of the bonded resin–dentin interface still limits the longevity of restoratives [1,2,3,4]. The major degradation in the interface is thought to be based on both enzymatic breakdown and hydrolysis of the collagen fibrils [5,6] and the polymerized resin matrix [7], which can undermine the adhesion between the tooth and composite and can lead to restoration failure. However, use of natural cross-linkers to stabilize dentin collagen and enhance dentin collagen’s resistance to degradation has the potential to improve the lifespan of composite restorations that are bonded to dentin [8,9,10,11,12].

Proanthocyanidin (PA), a polyphenolic natural component with many botanical sources, has been shown to be a natural collagen cross-linker, as well as a collagenase/matrix metalloproteinase (MMP) inhibitor [9,13,14]. It has been established that PA-rich grape seed extract (GSE) is an efficient collagen cross-linker even when treatment time is very short (<30 s) [8,15]. In previous publications, GSE was incorporated into a model adhesive, used as a priming agent, and incorporated into a phosphoric acid etchant. These studies showed that it increased dentin collagen’s resistance to degradation by bacterial collagenase [11,16,17]. From a clinical standpoint, the results of these studies on GSE are very promising. However, there are a large variety of other natural polyphenol-based extracts and synthetic cross-linkers available that need to be further evaluated with clinically relevant application times.

One alternative to GSE that should be evaluated is green tea extract (GTE), which contains polyphenolic compounds based on catechins, such as (−)-epigallocatechin-3-gallate (EGCG), (−)-epigallocatechin (EGC), and (−)-epicatechin-3-gallate (ECG) [18]. GTE could provide a viable alternative to GSE because GTE has been shown to inhibit matrix metalloproteinases [19], which are activated by acid etching [20]. GTE is also lighter in color than GSE, and because GSE stains demineralized dentin with a reddish-brown color [16], GTE has the potential to stain demineralized dentin with a more clinically acceptable color. Other alternatives to GSE are cranberry proanthocyanidins, which contain a unique A-linkage structure [21,22]. In a previous study, the interaction between cranberry juice extract (CJE) and demineralized dentin collagen was explained as “very weak” [23]; however, the PA concentrations of cranberry extract and the final prepared treatment solution used in the study were less than 1% and 0.001%, respectively, which may be a possible reason for the unexceptional results. Other than that study, it appears that little work has been done with regard to cranberry PA with A-linkage on demineralized dentin collagen’s cross-linking, MMP inhibition, and resistance to degradation. Given the substantial effects of the B-type PA in grape seed extract on demineralized dentin collagen, the effects of the A-type PA in cranberry juice extract also need to be evaluated.

Because dentin collagen’s stabilization and resistance to degradation can be accomplished by cross-linking [24,25], researchers have put in very much effort into exploring synthetic chemical cross-linkers, such as glutaraldehyde and carbodiimide [14,26,27,28]. Glutaraldehyde, a well-known collagen cross-linker, has been shown to increase dentin resistance to enzymatic degradation [29]. However, glutaraldehyde has drawbacks, especially with respect to its cytotoxicity [14] and slow chemical cross-linking reactions [16], which make it clinically inapplicable. N-(3-dimethylaminopropyl)-N’-ethylcarbodiimide hydrochloride (EDC) has also been shown to be a zero-length collagen cross-linker [30] that activates the carboxyl groups in amino acids and cross-links collagen peptides without creating extra linkage groups. With the addition of N-hydroxysuccinimide (NHS) to EDC, a coupling reaction takes place, and this has been shown to greatly prevent the hydrolysis of activated carboxyl groups and enhance the number of cross-links in the collagen matrix [26,31]. EDC/NHS also results in much lower cytotoxicity when compared to glutaraldehyde [25]. One study showed an increase in the biomechanical properties of EDC/NHS-treated dentin [26]. However, the treatment time was 1 h, which is clinically impractical. In another study, EDC was shown to inhibit dentin matrix metalloproteinases when applied as a primer for 1 min [32]. Nevertheless, EDC was not rinsed off after the 1 min application and was only followed by gentle air drying, which technically continued to promote MMP deactivation until the MMP inhibition test. It is still unknown how quickly EDC can interact with collagen or inhibit MMPs. In this study, dentin collagen treated with EDC/NHS was further evaluated by using a clinically relevant application time and compared to dentin collagen treated with natural polyphenol-rich sources.

The purpose of this study was to use a clinically relevant setting to evaluate the ability of natural sources that are rich in polyphenols to stabilize and protect the dentin collagen matrix from digestion by bacterial collagenase, to inhibit MMP activity in collagen. Specifically, the effects of green tea and cranberry juice extracts were evaluated and compared to those of grape seed extract. The synthetic collagen cross-linker EDC/NHS was further evaluated and compared to the natural sources that were rich in polyphenols. The experimental setting included a short treatment time (30 s), which is clinically practicable, and ultra-thin (10 µm) dentin films that were used to simulate the demineralized dentin layer after a total etching. The tested null hypothesis was that the resources of the tested cross-linkers had no effects on the collagen’s stabilization and MMP activities.

## 2. Materials and Methods

### 2.1. Reagents

Four collagen cross-linkers—grape seed extract (GSE), green tea extract (GTE), cranberry juice extract (CJE), and 1-ethyl-3-(3-dimethylaminopropyl) carbodiimide in combination with N-hydroxysuccinimide (EDC/NHS)—were used in this study. All treatment solutions, including the cross-linking solutions and control solution, were prepared with 0.96% phosphate buffered saline (PBS) solution according to our previous studies [9]. The concentrations of the polyphenols were kept the same among the natural cross-linking solutions based on the polyphenol percentage reported by manufacturers (Table 1). Because one of the major components in green tea extract is insoluble cellulose, the GTE solution was centrifuged and only the supernatant was used. The pH of the CJE solution was adjusted to near neutrality with NaOH to minimize any pH effects. The chemical cross-linker 0.3 M EDC/0.12 M NHS solution was prepared according to the literature [26].

Chemical reagents were purchased from Sigma-Aldrich (St. Louis, MO, USA). Formulations of reagents were based on those of previous studies [9,10,11]. Collagenase (from *Clostridium histolyticum*, type I, ≥125 U/mg) solution was made at 0.1% (*w*/*v*) in TESCA buffer (pH = 7.4, 0.36 mM CaCl_2_, 50 mM N-tris (hydroxymethyl) methyl-2-aminoethanesulfonic acid).

### 2.2. Dentin Film Preparation

Non-carious, un-erupted human third molars were collected without associated patient identifiers, and the collection protocol was determined not to be human subject research (NHSR 12-50) by the University Adult Heath Sciences Institutional Review Board. The teeth were stored in 0.96% PBS containing 0.002% sodium azide at 4 °C before use. Roots of teeth were removed 2–3 mm below the cementoenamel junction using a water-cooled low-speed diamond saw (Buehler, Lake Bluff, IL, USA). The remaining tooth was then attached to an aluminum disc by using cyanoacrylate adhesive (Zapit, Dental Ventures of America, Corona, CA, USA). The occlusal 1/3 of the crown and surrounding enamel was removed to result in a dentin block. The 10-µm-thick dentin films were cut from the superficial dentin portion on the block using a microtome (Leica SM2500S, Deerfield, IL, USA). Fifty dentin films were acquired from each tooth for a total of around 400 films with a size of approximately 6 mm × 5 mm when pooled together. The films were stored at 4 °C in 0.96% PBS containing 0.002% sodium azide.

### 2.3. Dentin Collagen Film Cross-Linking Treatments

The cross-linking treatment procedures were described in previous publications [9,17]. Sixty dentin films were randomly selected and divided into 6 specimens per treatment group (10 films per specimen, total 6 of specimens per treatment). Dentin films of each specimen were demineralized with 10% phosphoric acid for 30 min and rinsed with distilled water. Demineralized dentin films were then individually unrolled with a brush on a plastic coverslip. After excess water on the film was removed, a small drop of cross-linking solution or PBS was applied to cover the whole film for 30 s. The treated films were then rinsed with distilled water to completely remove residual treatment solution and were spread on plastic coverslips for air drying overnight. After drying, the treated films were subsequently analyzed with FTIR (*n* = 6 per treatment group), evaluated for their collagen stability via weight loss (WL) and hydroxyproline (HYP) release assays (*n* = 60 in each group), and analyzed for their collagen endogenous enzymatic activities via in situ zymography (*n* = 3/group).

### 2.4. FTIR Analysis of Demineralized Dentin Films

Six treated and dried dentin collagen films per group were submitted on a BaF_2_ disc (Reflex Analytical Corporation, Ridgewood, NJ, USA) to a Fourier-transform infrared spectroscopy (FTIR) examination (Spectrum One, Perkin Elmer, Waltham, MA, USA) to analyze the collagen cross-linking effects of the natural extracts (GSE, GTE, CRE) and EDC/NHS via the chemical interactions. Each FTIR spectrum was collected at a resolution of 4 cm^−1^ and a scan number of 128 in the 1750–950 cm^−1^ wavenumber range. The integration area was calculated for peaks at 1550 (amide II), 1450 (CH_2_ scissoring), and 1400 cm^−1^ (C–O bending) using the Spectrum software (Perkin Elmer, Waltham, MA, USA) after a two-point baseline adjustment, and the peak area ratios of A1550/A1450 and A1400/A1450 were determined.

### 2.5. Weight Loss Measurement of Dentin Collagen’s Biostability against Collagenase Degradation

The 6 dried specimens per treatment group were weighed with a Mettler Toledo AG285 Analytical Balance (Columbus, OH, USA, d = 0.01/0.1 mg), then digested at 37 °C for 1 h with 300 µL of 0.1% bacterial collagenase. The liquid digests were then recovered from each specimen and frozen at −20 °C prior to hydroxyproline analysis. After digestion, the remaining films of each specimen were dried overnight after rinsing and then weighed again. The whole weighing procedure for each specimen was treatment–weighing–digestion–weighing. The percentage weight loss (WL) of each specimen was calculated with regard to the weight of the films after digestion.

### 2.6. Quantitation of Hydroxyproline Content Released from the Digested Dentin Collagen Films

The digested collagen solutions from the dentin collagen films were thawed, hydrolyzed with 6 M HCl at 110 °C for 24 h, and then dried in a vacuum desiccator over NaOH pellets. The hydroxyproline content of the digests was analyzed as described below. Briefly, the dried hydrolysates were re-dissolved with 125 µL of distilled water, mixed with 125 µL of 0.05 M CuSO_4_ and 125 µL of 2.5 M NaOH, and heated for 5 min at 40 °C prior to oxidation with 125 µL of 6% H_2_O_2_ for 10 min at 40 °C. Colorimetric detection was achieved through the addition of 500 µL of 1.5 M H_2_SO_4_ and 250 µL of 5% dimethylamino benzaldehyde dissolved in n-propanol followed by heating for 16 min at 70 °C. After cooling, 125 µL of each sample was transferred to a 96-well microplate (Corning, NY, USA) and read at 555 nm by using a Biotek Epoch Microplate Spectrophotometer (Biotek Instruments, Winooski, VT, USA). The hydroxyproline content of unknowns was calculated from a standard curve constructed with a similar procedure with 0–200 µg of trans-4-hydroxy-L-proline, which was expressed as µg/mg collagen film. The hydroxyproline release contents of the six specimens (*n* = 6) in each treatment group were averaged for a statistical analysis.

### 2.7. Scanning Electron Microscopy (SEM) and Transmission Electron Microscopy (TEM) Analysis

The dentin slabs prepared for the cross-linking treatments were used to observe the morphological changes in collagen before and after the digestion process by using scanning electron microscopy (SEM) and transmission electron microscopy (TEM). After removal of the crown, a uniform smear layer was generated by means of wet 600-grit silicon carbide paper (Buehler, Lake Bluff, IL, USA) on the exposed dentin surface. The dentin was then cut in 1.5 mm (for SEM) or 0.5 mm (TEM) increments in the occlusal–apical direction, and then cut approximately 1.5 mm parallel to the surface. Slabs used for SEM were notched in the midpoint of the pulpal surface for subsequent fracturing.

The slabs were then acid etched for 15 s with 32% H_3_PO_4_ gel (Scotchbond Universal Etchant, 3M ESPE, St. Paul, USA) and rinsed with distilled water for 10 s. Four demineralized dentin slabs from the same tooth were treated with the respective treatment solution for 30 s with the same treatment procedure as above. Of the four slabs prepared for each treatment group, two were selected for addition of 1 mL of 0.96% PBS at 37 °C for 1 h to be compared with the other two slabs, which underwent digestion with 0.1% collagenase solution at 37 °C for 1 h.

The notched slabs for SEM were fixed in 2.5% glutaraldehyde in 0.1 M sodium cacodylate buffer for 30 min and dehydrated in increasing concentrations of ethanol (33%, 67%, and 85%) for 15 min each, followed by concentrations of 95% and 100% for 30 min each. The slabs were then dried, fractured, mounted on an aluminum stub with the fractured face up, and coated with carbon. The collagen layer on the fractured surface was examined at 5 kV in an FEI/Philips XL30 Field-Emission Environmental SEM (Philips, Eindhoven, Netherlands) at a variety of magnifications.

The un-notched slabs were first fixed for 1.5 h using 1% osmium tetroxide and then underwent the dehydration process using the graded solutions of ethanol mentioned above. These slabs were subsequently immersed in 1:1 solution of ethanol and propylene oxide (PO) for 30 min and then 100% PO for 120 min, followed by a 1:1 solution of PO and epoxy resin (Embed-812, Electron Microscopy Sciences, Hatfield, PA, USA) for 24 h. Following the final embedding in pure epoxy resin, slabs were incubated for 48 h at 60 °C in an oven. Ultra-thin (90-nm-thick) sections were cut using an EM-UC7 ultramicrotome (Leica, Buffalo Grove, IL, USA). After staining with 1% phosphor-tungstic acid, the morphology of the dentin collagen was examined at an 80 kV accelerating voltage in a CM12 electron microscope (FEI, Hillsboro, OR, USA).

### 2.8. MMP Activity in Dentin Matrix Collagen with a Confocal Laser Scanning Microscope (CLSM)

Three dentin collagen films from each treatment group, as well as three untreated films (negative control), were utilized for analysis of the activity of MMPs 2 and 9 within the dentin matrix collagen. A fluorescein-conjugated gelatin (E-12055, Molecular Probes, Eugene, OR, USA) was made by following the manufacturer’s protocols directly before usage. Immediately after the cross-linking treatments (for 30 s), the collagen films were thoroughly rinsed with water and spread on glass microscope slides. Then, the gelatin was dropped (3 µL) on a collagen film and incubated in a chamber (humidity of 90%) that was shielded from room light for 24 h at 37 °C. The endogenous MMP activity within the collagen films relied on hydrolysis of the quenched gelatin. Each microscope slide containing the films was covered with a coverslip and visualized with a confocal laser scanning microscope (CLSM) (TCS SP5 II, Leica Microsystems, Buffalo Grove, IL, USA) in a fluorescence mode (40 × objective lens of 0.95 NA) at 488 nm of excitation and 530 nm of emission. Three images obtained from the same z layer were randomly captured for each film. The fluorescence intensities released by the hydrolyzed fluorescein-conjugated gelatin of all images (*n* = 9 images for each group) were quantified using the NIH Image J 1.8.0 software (Bethesda, MD, USA) [33]. The extent of the endogenous gelatinolytic activity was characterized by the percentage of green fluorescence intensity radiated within the treated collagen films as compared to the untreated control (100%).

### 2.9. Statistical Analysis

The results from FTIR peak ratios, percent weight loss and hydroxyproline content, and in situ MMP zymography are expressed as mean ± standard deviation (SD). The Shapiro–Wilk and Kolmogorov–Smirnov tests were employed to assess whether the data followed a normal distribution. Levene’s test was performed to determine the homogeneity of variance. Data were assessed using a one-way analysis of variance (ANOVA) followed by Tukey’s post-hoc test by using IBM SPSS v23 (IBM SPSS Inc., Chicago, IL, USA). Statistical significance was set at 0.05.

## 3. Results

### 3.1. FTIR Spectroscopic Analysis

**Figure 1:**
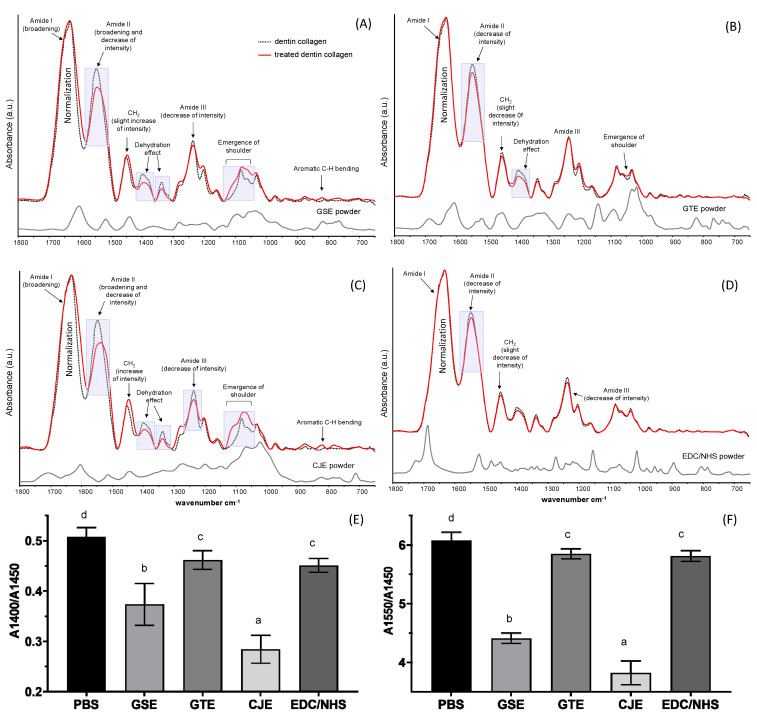
Representative FTIR spectra of dentin collagen before (black dotted line) and after cross-linker treatment (solid red line) for 30 s, as well as cross-linker powders (solid black line). (**A**) Treated with GSE, (**B**) treated with GTE, (**C**) treated with CJE, and (**D**) treated with EDC/NHS. The untreated and treated collagen spectra were normalized based on the intensity of amide I peak (**A**–**D**). Spectral changes and peak assignments were labeled with more obvious changes that are highlighted in the shaded rectangular areas. (**E**) Peak ratios of C-O and C-N bending to CH_2_ (A1400/A1450) and (**F**) peak ratios of amide II to CH_2_ (A1550/A1450). Means with different letters are significantly different (*p* < 0.05). PBS: phosphate buffered saline. GSE: grape seed extract. GTE: green tea extract. CJE: cranberry juice extract. EDC/NHS: carbodiimide/n-hydroxysuccinimide.

Figure 1 presents a comparison of the FTIR spectra of dentin collagen films with or without cross-linking treatments and cross-linkers. Peaks that were characteristic of collagen that have been well documented in the literature were assigned—specifically, the amide I peak for the peptide C=O stretching at ~1633 cm^−1^, the amide II peak for the in-plane N–H bending and C–N stretching at ~1550 cm^−1^, the amide III peak for the C–N, C-H, and N–H deformation at 1240 cm^−1^, C–O and C–N bending at 1400 cm^−1^, and the CH_2_ scissoring at ~1450 cm^−1^. Some of the cross-linker peaks overlapped with the collagen peaks and could, therefore, not be recognized. However, a careful look at the FTIR spectra of the cross-linker-treated films showed the evident spectral alterations and clear incorporation of GSE and CJE into the demineralized dentin specimens after a thorough water rinse (Figure 1A,C). For example, the broadening of amide I (~1633 cm^−1^), right shoulder formation and intensity decrease of amide II (~1550 cm^−1^), decrease in intensity at ~1400 cm^−1^ and amide III, and shoulder emergence between ~1100 and 1000 cm^−1^ were manifestly recognized after 30 s of treatment with GSE and CJE. On the other hand, these spectral alterations were largely reduced in the GTE-treated group (Figure 1B) or almost unnoticeable in the group treated with EDC/NHS (Figure 1D). Quantitatively, the A1400/A1450 and A1550/A1450 peak ratios (Figure 1E,F) of the specimens treated with CJE were the lowest among the groups, followed by the GSE-treated group and then the GTE- and EDC/NHS-treated groups, whose ratios became marginally—but still significantly—lower than that of the control (*p* < 0.05).

### 3.2. Weight Loss and Quantitation of Hydroxyproline Release from the Digested Collagen Films

**Figure 2:**
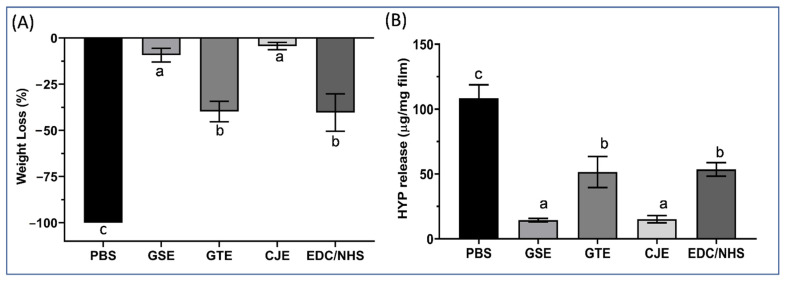
(**A**) Percent weight loss of the untreated PBS control and cross-linker-treated (for 30 s) demineralized dentin films after digestion with 0.1% collagenase solution for 1 h. Means with different letters are significantly different (*p* < 0.05). (**B**) Hydroxyproline (HYP) release content of the untreated PBS control and cross-linker-treated (for 30 s) demineralized dentin films after digestion with 0.1% collagenase solution for 1 h. Means with different letters are significantly different (*p* < 0.05). PBS: phosphate buffered saline. GSE: grape seed extract. GTE: green tea extract. CJE: cranberry juice extract. EDC/NHS: carbodiimide/n-hydroxysuccinimide.

After bacterial collagenase digestion, the weight loss of the PBS control and cross-linker-treated collagen films is shown in Figure 2A. The PBS control films were totally consumed after digestion for 1 h. In contrast, the collagen films treated with different cross-linkers displayed varying degrees of resistance to collagenase digestion compared to the PBS-treated films. Among the cross-linkers, CJE and GSE significantly enhanced resistance to degradation compared with GTE and EDC/NHS.

To confirm the resistance of collagen to exogenous collagenase digestion, the total amount of hydroxyproline released from the digested dentin films was measured and is presented as micrograms per milligram of collagen in the digestant solutions (Figure 2B). The amount of HYP released from the cross-linker-treated collagen groups was significantly reduced in comparison to that from the PBS control group. Among the cross-linkers, the hydroxyproline released from the CJE and GSE groups was drastically decreased compared with that released from the GTE and EDC/NHS groups. When ranked from the lowest to the highest, the collagen’s resistance to collagenase digestion was in following order: PBS < EDC/NHS = GTE < GSE = CJE.

### 3.3. SEM

**Figure 3:**
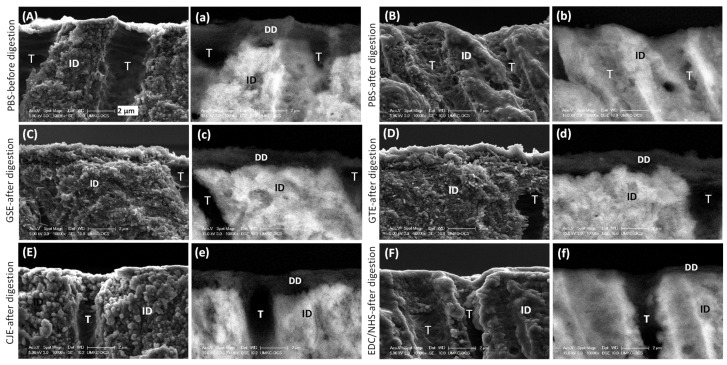
Representative SEM images of the untreated PBS control and cross-linker-treated demineralized dentin layers before and after 1 h of digestion in 0.1% collagenase in the secondary electron mode (**A**–**F**) and the backscattered electron mode (**a**–**f**). (**A**,**a**) PBS control before digestion. (**B**,**b**) PBS control after digestion. (**C**,**c**) GSE-treated group after digestion. (**D**,**d**) GTE-treated group after digestion. (**E**,**e**) CJE-treated group after digestion. (**F**,**f**) EDC/NHS-treated group after digestion. PBS: phosphate buffered saline. GSE: grape seed extract. GTE: green tea extract. CJE: cranberry juice extract. EDC/NHS: carbodiimide/n-hydroxysuccinimide. DD: demineralized dentin. ID: intact dentin. T: tubule.

The cross-sections of the demineralized dentin (DD) layers before digestion all showed distinct collagen fibrils (SEM mode) and dark layers (BSEM mode) (Figure 3). After digestion, the demineralized layer of the PBS control group was totally removed (Figure 3B). The demineralized layers of the GSE-, GTE-, and CJE-treated groups were still evident and largely unchanged after collagenase treatment (Figure 3C–E). The EDC/NHS-treated group’s demineralized dentin layer was mostly removed by the collagenase treatment (Figure 3F).

### 3.4. TEM

**Figure 4:**
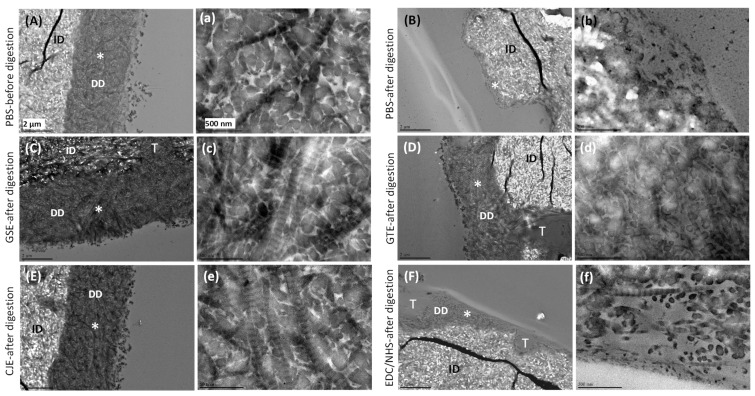
(**A**–**F**) Representative TEM images of the untreated PBS control and cross-linker-treated demineralized dentin layers before and after 1 h of digestion in 0.1% collagenase. (**a**–**f**) Representative high-magnification view of the top demineralized dentin layer from the same upper-case-letter figure in the area labeled as *. (**A**,**a**) PBS control before digestion. (**B**,**b**) PBS control after digestion. (**C**,**c**) GSE-treated group after digestion. (**D**,**d**) GTE-treated group after digestion. (**E**,**e**) CJE-treated group after digestion. (**F**,**f**) EDC/NHS-treated group after digestion. PBS: phosphate buffered saline. GSE: grape seed extract. GTE: green tea extract. CJE: cranberry juice extract. EDC/NHS: carbodiimide/n-hydroxysuccinimide. DD: demineralized dentin. ID: intact dentin. T: tubule.

The TEM images of acid-etched dentin slabs before digestion displayed an obvious DD layer (Figure 4A) in which the collagen fibers displayed the typical 67 nm banding pattern (Figure 4a). For the untreated PBS control, this layer completely degraded after collagenase digestion (Figure 4B,b). TEM images of the cross-linker-treated demineralized dentin layers after collagenase digestion are shown in Figure 4C–F. In particular, the morphology of the collagen fibrils within regions of the demineralized dentin layer was also examined under higher magnification (Figure 4c–f). The GSE (Figure 4C,c), GTE (Figure 4D,d), and CJE (Figure 4E,e) groups displayed densely packed fibrils within the DD layer, and the collagen fibrils exhibited the typical banding pattern of type I collagen, except for GTE (Figure 4d), in which the banding pattern was not evident. However, the EDC/NHS-treated demineralized dentin layer showed thin and loosely scattered fibrils and voids after the collagenase treatment (Figure 4F,f).

### 3.5. MMP Activity in the Dentin Collagen Matrix with Confocal Laser Scanning Microscopy

**Figure 5:**
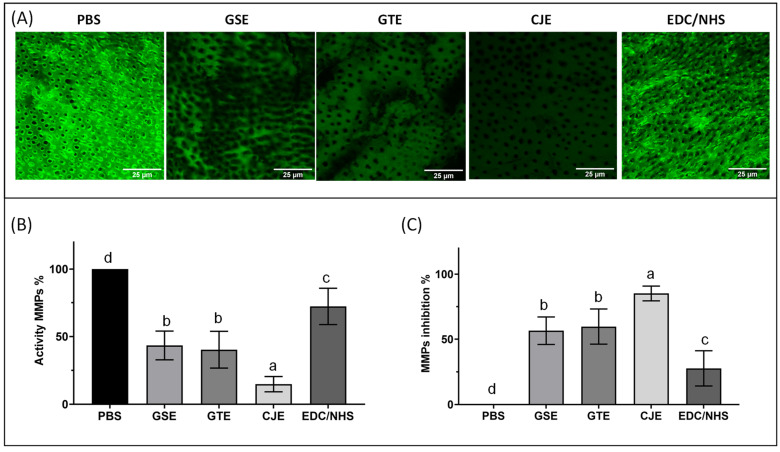
Representative in situ zymogram confocal laser scanning microscopic (CLSM) images acquired in the green channel showing the fluorescence attributed to the MMP activity in the dentin collagen films for all experimental groups after 24 h of incubation in the quenched fluorescein-labeled gelatin (**A**). The graphs show the quantitative MMP activity (**B**) and MMP inhibition (**C**) for all groups. Means with different letters are significantly different (*p* < 0.05). PBS: phosphate buffered saline. GSE: grape seed extract. GTE: green tea extract. CJE: cranberry juice extract. EDC/NHS: carbodiimide/n-hydroxysuccinimide.

The endogenous MMP activities within the collagen films were examined using confocal laser scanning microscopy and in situ zymography; representative images and the green fluorescence intensities representing endogenous MMP activity or the inhibition of the untreated and cross-linker-treated collagen films are presented in Figure 5. In the PBS control films without cross-linker treatment, robust and constant fluorescence indicated the strongest gelatinolytic (MMPs) activity. All cross-linker-treated groups showed reduced MMP activities. The fluorescence intensities (or MMP activity) of the collagen films were reduced by ~60% when treated with GSE or GTE, and the difference in activity between these two was not significant (*p* > 0.05). When CJE was used, the green fluorescence intensity of the collagen films was dramatically reduced (by ~85%), indicating nearly complete inhibition of gelatinase activity. The EDC/NHS treatment only resulted in a slightly reduced gelatinolytic activity in the films that was only ~28% less intense than in the PBS control. When ranked from the strongest to the weakest, the MMP activity was in following order: PBS > EDC/NHS > GSE = GTE > CJE.

## 4. Discussion

The purpose of this study was to evaluate the effects of cross-linker treatments on dentin collagen’s stabilization and MMP activity in a clinically relevant situation, where the treatment time must be short (in tens of seconds rather than minutes) and the demineralized collagen layer to be treated is only several microns thick. Common practices for cutting dentin into beams with sizes on the millimeter scale or for grinding dentin into powders with a mesh size of 20–40 (400–840 µm) [34] need to be ruled out because the homogeneous diffusion of a cross-linker into these thick samples is hard to achieve within such a short treatment time, which could cause the experimental result to be misrepresented. In addition, the grinding process may cause alterations in the collagen’s structure. To better mimic the thickness and structure of the acid-etched dentin layer in a clinical setting, ultra-thin dentin films (10 µm) were used for cross-linking treatments of 30 s. The use of thin films not only permits uniform infiltration of the cross-linker solution into the entire specimen, but also enables the quick and complete removal of the loosely bound cross-linker from the spongy matrix, thus ensuring the accurate comparison of different cross-linkers.

After 30 s of cross-linking of the demineralized dentin films, all treatment groups exhibited a significantly increased resistance to degradation by exogenous bacterial collagenase and endogenous MMP inhibition of collagen compared with the PBS control. Among the treatment groups, the CJE- and GSE-treated groups were more resistant to collagenase digestion than the GTE- or EDC/NHS-treated groups (*p* < 0.05). The results were generally in agreement with the cross-linking efficacy of the four cross-linkers revealed through FTIR and the cross-linkers’ in situ performance on the acid-etched layer of dentin substrates revealed through SEM/TEM. The collagen films treated with CJE showed the lowest MMP activity (or highest MMP inhibition), followed by the films treated with GSE and GTE, then EDC/NHS. Thus, the null hypothesis was rejected.

The FTIR spectra of the collagen with and without treatment were closely compared to identify the detailed interactions between the cross-linkers and collagen. As seen in Figure 1, spectral changes were generally more manifest in the CJE- and GSE-treated groups, while they were less noticeable in the GTE-treated group and almost negligible in the EDC/NHS-treated group when compared to the untreated control. In comparison with the spectra of the three polyphenols used, certain spectral changes were from the incorporation of these cross-linkers in the collagen matrix after extensive solvent rinsing. For example, the emergence of shoulder (1100–1000 cm^−1^) and aromatic C–H bending (900–750 cm^−1^) peaks could have corresponding parts in the spectra of polyphenol powders, with the CJE- and GSE-treated groups showing higher levels of incorporation or interaction and the GTE-treated group showing a lower amount. Other spectral changes might be associated with the following potential interactions. For example, one likely interaction was assigned to the dehydration of collagen induced by cross-linking (due to the “hydrophobic effects” of polyphenols [35]), which was demonstrated by a decrease in intensity at ~1400 cm^−1^ [9,36]; as stated by Miles et al., dehydration offers a solid indication of cross-linking and is key cause of improved collagen stability [9,36]. In addition, the amide I (~1633 cm^−1^) broadening and amide II (~1550 cm^−1^) intensity decrease and shoulder formation were assigned to hydrogen bonding via the phenolic hydroxyl (OH) groups of natural compounds and amino or amide groups of collagen [37], which is considered as another main polyphenol/collagen interaction mechanism [37,38]. Furthermore, collagen cross-linking caused a decrease in –NH_2_, which was reflected by an intensity drop in the amide II peak [39,40]. The extent of cross-linking-induced spectral changes was quantitatively measured with the A1400/A1450 and A1550/A1450 ratios (Figure 1E,F). The lower the ratios, the higher the collagen/cross-linker interactions. The CJE-treated group showed the lowest ratios while the GTE-treated group showed the highest ratios among the polyphenols.

Differently from three non-zero-length polyphenol cross-linkers, EDC/NHS is considered a zero-length cross-linker, which achieves collagen cross-linking without the introduction of extra atoms or a spacer. EDC is used to activate the carboxylic acid groups of collagen and to mediate the amide linkage formation between carboxylates and amines among the collagen molecules with no intervening linker. The addition of NHS prevents the hydrolysis of activated carboxyls and effectively increases the number of cross-links in the collagen matrix [41]. The negligible spectral changes between the untreated and EDC/NHS-treated collagen (Figure 1D) proved that there was no incorporation of EDC/NHS into the collagen. To reveal any possible structural changes caused by cross-linking, the spectral peak ratios of A1400/A1450 and A1550/A1450 were calculated. The two ratios were slightly but significantly reduced as compared to the control, indicating that the intensities of 1400 cm^−1^ and amide II were relatively decreased (Figure 1E,F). The spectral changes could be attributed to the additional formation of amide linkages between carboxylates and amines among the collagen molecules after cross-linking.

It is of interest to study if dentin collagen could become resistant to degradation within a clinically relevant time (30 s) after cross-linking. Exogeneous bacterial collagenase was used to accelerate the collagen digestion process. Interestingly, the treated collagen specimens’ digestion behavior (WL/HYP) generally corresponded well with the spectral peak ratios from FTIR, showing a close correlation between the degradation resistance and the extent of interactions between the collagen and cross-linker. For polyphenols, the inhibition of exogenous collagenase has been attributed to the hydroxyl groups of the polyphenols and their ability to act as a hydrogen bond donor/acceptor with collagenase [42]. Hydrophobic interactions of the benzene ring of the polyphenols also play a role in the inhibition of collagenase [42]. The greater the interactions of polyphenols with dentin collagen are, the higher the inhibition of exogenous collagenolytic enzymes will be. For zero-length EDC/NHS, degradation resistance only relies on the formation of additional amide bonds with changes in the conformation of collagen, which makes the collagen structure resistant to collagenase.

To mimic a clinically relevant condition, a dentin surface covered with a smear layer was acid etched for 15 s with H_3_PO_4_ to generate a several-micron-thick demineralized layer followed by priming treatment for 30 s with a cross-linker, and it was subsequently subjected to collagenase digestion after rinsing off the un-bound cross-linker in the layer with water. Indeed, the water rinse was applied to both demineralized layers and films after the cross-linking treatment to ensure the removal of residual cross-linkers that were un-bonded with collagen. This rinsing process would not only stop the cross-linking reactions, but also confirm that the collagen’s resistance to digestion by cross-linkers was due to irreversible cross-linking reactions, not by cross-linker residuals. The SEM/TEM results (Figure 3 and Figure 4) indicated that all cross-linkers could infiltrate into the demineralized layer and stabilize the collagen against degradation within a fast, clinically relevant time (30 s), except for EDC/NHS. The DD collagen layer and periodicity in the GSE- and CJE-treated groups appeared intact after digestion, while a slight degradation and loss of collagen periodicity was seen in the GTE-treated group, which was in agreement with the above quantitative outcomes on the films. The periodicity or characteristic banding pattern of collagen resulted from the overlaying of the gap and overlap zones. C- and N-terminal telopeptides were located in the overlap zone; consequently, their cleavage by collagenase caused the damage to the collagen banding pattern (Figure 4d).

The endogenous MMP results revealed that the 30 s cross-linking treatment of the dentin collagen films reduced the gelatinolytic activity for CJE by ~85%, for GSE/GTE by ~60%, and for EDC/NHS by ~28%, which was the lowest inhibition among the four cross-linkers. The quantitative results of the endogenous gelatinolytic activity (MMPs) and the resistance of collagen to digestion by exogenous collagenase (WL/HYP) were closely compared for different cross-linkers (Figure 2 and Figure 5). The MMP and WL/HYP data were directly correlated for the CJE- and EDC/NHS-treated groups, with the former showing the lowest MMP activity and WL/HYP values and the latter showing the highest MMP activity and WL/HYP values among the treated groups. However, these data were not directly correlated for the GSE- and GTE-treated groups. The GSE-treated group showed one of the lowest WL/HYP values (comparable to that of the CJE-treated group), but its MMP activity value was much higher than that of the CJE-treated group. Similarly, the GTE-treated group showed one of the highest WL/HYP values (similar to that of the EDC/NHS-treated group), but its MMP value was lower than that of the EDC/NHS-treated group and close to that of the GSE-treated group. These discrepancies might be attributed to the different measures/origins between collagenase degradation and MMPs, which show dissimilar digestion patterns. In comparison with host-derived, matrix-bound MMPs, bacterial collagenases have very broad recognition sites that dissociate/cleave the collagen at wide-ranging spots in the triple helix [43]. Although a bacterial collagenase is a great model for fast collagen digestion, it would be irrelevant for clinical situations because bio-degradation by endogenous MMPs more closely represents the biochemical process in vivo. It would be worth studying if high deactivation of MMPs by cross-linkers would translate to increased long-term collagen bio-stability under clinical conditions.

As described above, although GSE is capable of rapidly stabilizing the demineralized dentin layer [16], the incorporation of GSE into a protocol for a composite restoration has had setbacks. The addition of PA-rich GSE into a phosphoric acid etchant was shown to increase dentin collagen’s resistance to degradation, but required a decreased phosphoric acid concentration or a longer etching time [17]. The incorporation of GSE into an adhesive showed a reduced degree of monomer conversion [11]. The application of GSE as a primer has been shown to improve dentin collagen’s bio-stability against digestion and is very promising given the short treatment time [16]. However, the negative impact of a priming agent is the additional step and time required for subsequent rinsing. In addition, there is a concern that collagen’s resistance to degradation via GSE priming would not last long due to potential breakdown of B-type linkage in GSE [44,45]. This has led to the exploration of viable alternatives to GSE that may show similar or better results than GSE on demineralized dentin collagen without the above setbacks.

Dentin collagen films treated with GTE displayed less MMP activity and are more resistant to digestion by bacterial collagenase than the untreated control, which confirmed the findings of previous studies on the role of GTE in the inhibition of collagenolytic activity [42]. Although GTE showed a reduced MMP activity similar to that of GSE, it did not affect collagen digestion to the same extent as GSE. GTE does appear to present a viable option in regard to the color with which it stains demineralized dentin. The color of the films after the GTE treatment was similar to that of dentin before the treatment. It would be interesting to see if the treatment of dentin collagen with GTE could be improved by increasing its concentration, as well as by increasing the treatment time.

Although CJE and GSE were not significantly different from each other in terms of the WL/HYP measurements, the MMP activity (15%) of demineralized dentin films treated with CJE was much less than that of those treated with GSE (43%), which was the lowest of all of the groups. The difference in MMP activity may be due to the structural difference between the A-linkage PA in CJE [21,22] and B-linkage PA in GSE [22]. Previously, the interaction between CJE and demineralized dentin collagen was explained as “very weak” due to the very low PA content [23]. However, by using a CJE treatment solution that contained 0.65% PA, the interaction of CJE and demineralized dentin collagen was quite robust. The effectiveness of CJE as an MMP inhibitor was evidenced by the dramatically reduced matrix-bound enzymatic activity, which almost completely inactivated the gelatinolytic activity in the collagen films after 30 s of treatment. It has been reported that A- linkage PA is more hydrolytically stable than B-linkage PA, thus favoring long-term stability [45,46]. Given the results of the better MMP inhibition of CJE than that of GSE, it would be interesting to determine which treatment (GSE or CJE) is better than the other under in vivo conditions during a long-term durability test. Further work is also needed to test the incorporation of A-type PA from CJE into a model adhesive or into a phosphoric acid etchant.

After the treatment of demineralized collagen with EDC/NHS for 30 s, both the collagen inhibition to matrix-bound MMPs and the resistance to degradation by bacterial collagenase were improved compared to the untreated control. However, the application of EDC/NHS as a priming agent for 30 s did not increase collagen’s protection from gelatinolytic and collagenolytic enzymes as much as the application of GSE or CJE did. It is believed that the rate of chemical cross-linking of collagen via the formation of amide bonds in EDC/NHS is slower than that in polyphenols, whose interactions with collagen are instantaneous [16,47]. On the other hand, unlike polyphenols, which are free-radical scavengers and must be rinsed off after treatment, EDC/NHS does not exhibit an antioxidant nature or produce cytotoxicity because the byproduct generated after cross-linking is urea, which can be easily removed from substrates. In a separate experiment, when collagen films treated with EDC/NHS for 30 s were not rinsed off, the MMP activity was dramatically reduced by ~75% (24.5 ± 1.9%), as compared to the only ~28% reduction in the films that were treated in the same way, but with rinsing. Due to its non-antioxidant advantage over polyphenols, the direct incorporation of EDC/NHS into an adhesive deserves further investigation.

## 5. Conclusions

In summary, all treatment groups improved dentin collagen’s biological stability and resistance to degradation by exogenous bacterial collagenase. Dentin collagen treated with GSE or CJE for 30 s was more resistant to digestion than collagen treated with GTE or EDC/NHS under similar conditions. Moreover, the treated collagen resulted in a dramatic reduction in matrix-bound gelatinolytic enzyme activity compared to the untreated control (*p* < 0.05), with CJE-treated collagen films exhibiting significantly lower endogenous enzyme activity than the other cross-linkers (*p* < 0.05). Therefore, CJE may be a better alternative to GSE due to its higher MMP inhibition and more stable A-linkage; thus, it certainly deserves further evaluation as a clinically feasible approach to improving the longevity of dentin bonding in composite restorations.

## Figures and Tables

**Table 1:** Treatment solutions and their composition, manufacturer, and polyphenol content.

Solution	Composition (w/v)	Manufacturer	Polyphenol Content	pH
Control—PBS	0.96% PBS	Sigma Life Science, Dulbecco’s Phosphate Buffered Saline, St. Louis, MO, USA	N.A.	7.42
GSE	0.65% grape seed extract	Polyphenolics, MegaNatural Gold Grape Seed Extract, Madera, CA, USA	95%	6.84
GTE	1.3% green tea extract	True Fit Vitamins, Green Tea Extract, Bellevue, WA, USA	50%	6.60
CJE	4.2% cranberry juice extract	Ocean Spray, Cranberry Extract Powder, Lakeville-Middleboro, MA, USA	15.5%	3.10
EDC/NHS	0.3 M EDC 0.12 M NHS	Sigma-Aldrich, St. Louis, MO, USA	N.A.	5.91

## Data Availability

The data are available within the article and can be requested from the corresponding author.

## References

[1] Armstrong S.R., Vargas M.A., Chung I., Pashley D.H., Campbell J.A., Laffoon J.E., Qian F. (2004). Resin-dentin interfacial ultrastructure and microtensile dentin bond strength after five-year water storage. Oper. Dent..

[2] Breschi L., Mazzoni A., Ruggeri A., Cadenaro M., Di Lenarda R., Dorigo E.D.S. (2008). Dental adhesion review: Aging and stability of the bonded interface. Dent. Mater..

[3] De Munck J.D., Van Landuyt K., Peumans M., Poitevin A., Lambrechts P., Braem M., Van Meerbeek B. (2005). A critical review of the durability of adhesion to tooth tissue: Methods and results. J. Dent. Res..

[4] Pashley D.H., Tay F.R., Breschi L., Tjäderhane L., Carvalho R.M., Carrilho M., Tezvergil-Mutluay A. (2011). State of the art etch-and-rinse adhesives. Dent. Mater..

[5] Brackett W.W., Tay F.R., Brackett M.G., Dib A., Sword R.J., Pashley D.H. (2007). The Effect of Chlorhexidine on Dentin Hybrid Layers In Vivo. Oper. Dent..

[6] Hashimoto M., Tay F.R., Ohno H., Sano H., Kaga M., Yiu C., Kumagai H., Kudou Y., Kubota M., Oguchi H. (2003). SEM and TEM analysis of water degradation of human dentinal collagen. J. Biomed. Mater. Res..

[7] Hashimoto M., Ohno H., Sano H., Kaga M., Oguchi H. (2003). In vitro degradation of resin–dentin bonds analyzed by microtensile bond test, scanning and transmission electron microscopy. Biomaterials.

[8] Liu Y., Wang Y. (2013). Proanthocyanidins’ efficacy in stabilizing dentin collagen against enzymatic degradation: MALDI-TOF and FTIR analyses. J. Dent..

[9] Liu Y., Chen M., Yao X., Xu C., Zhang Y., Wang Y. (2013). Enhancement in dentin collagen’s biological stability after proanthocyanidins treatment in clinically relevant time periods. Dent. Mater..

[10] Hechler B., Yao X., Wang Y. (2012). Proanthocyanidins alter adhesive/dentin bonding strengths when included in a bonding system. Am. J. Dent..

[11] Green B., Yao X., Ganguly A., Xu C., Dusevich V., Walker M.P., Wang Y. (2010). Grape seed proanthocyanidins increase collagen biodegra-dation resistance in the dentin/adhesive interface when included in an adhesive. J. Dent..

[12] Hass V., Luque-Martinez I.V., Gutierrez M.F., Moreira C.G., Gotti V.B., Feitosa V.P., Koller G., Otuki M.F., Loguercio A.D., Reis A. (2016). Collagen cross-linkers on dentin bonding: Stability of the adhesive interfaces, degree of conversion of the adhesive, cytotoxicity and in situ MMP inhibition. Dent. Mater..

[13] Ferreira D., Slade D. (2002). Oligomeric proanthocyanidins: Naturally occurring O-heterocycles. Nat. Prod. Rep..

[14] Han B., Jaurequi J., Tang B.W., Nimni M.E. (2003). Proanthocyanidin: A natural crosslinking reagent for stabilizing collagen matrices. J. Biomed. Mater. Res..

[15] Liu Y., Wang Y. (2013). Effect of proanthocyanidins and photo-initiators on photo-polymerization of a dental adhesive. J. Dent..

[16] Liu Y., Dusevich V., Wang Y. (2013). Proanthocyanidins Rapidly Stabilize the Demineralized Dentin Layer. J. Dent. Res..

[17] Liu Y., Dusevich V., Wang Y. (2014). Addition of Grape Seed Extract Renders Phosphoric Acid a Collagen-stabilizing Etchant. J. Dent. Res..

[18] Khan N., Mukhtar H. (2013). Tea and health: Studies in humans. Curr. Pharm. Des..

[19] Demeule M., Brossard M., Pagé M., Gingras D., Béliveau R. (2000). Matrix metalloproteinase inhibition by green tea catechins. Biochim. Biophys. Acta (BBA) Protein Struct. Mol. Enzym..

[20] Nishitani Y., Yoshiyama M., Wadgaonkar B., Breschi L., Mannello F., Mazzoni A., Carvalho R.M., Tjaderhane L., Tay F.R., Pashley D.H. (2006). Activation of gelatinolytic/collagenolytic activity in dentin by self-etching adhesives. Eur. J. Oral Sci..

[21] Foo L.Y., Lu Y., Howell A.B., Vorsa N. (2000). A-Type Proanthocyanidin Trimers from Cranberry that Inhibit Adherence of Uropathogenic P-Fimbriated *Escherichia coli*. J. Nat. Prod..

[22] Howell A.B., Reed J.D., Krueger C.G., Winterbottom R., Cunningham D.G., Leahy M. (2005). A-type cranberry proanthocyanidins and uropathogenic bacterial anti-adhesion activity. Phytochemistry.

[23] Castellan C.S., Bedran-Russo A.K., Karol S., Pereira P.N.R. (2011). Long-term stability of dentin matrix following treatment with various natural collagen cross-linkers. J. Mech. Behav. Biomed. Mater..

[24] Nimni M.E., Cheung D., Strates B., Kodama M., Sheikh K. (1987). Chemically modified collagen: A natural biomaterial for tissue re-placement. J. Biomed. Mater. Res..

[25] Khor E. (1997). Methods for the treatment of collagenous tissues for bioprostheses. Biomaterials.

[26] Bedran-Russo A.K., Vidal C.M., Dos Santos P.H., Castellan C.S. (2010). Long-term effect of carbodiimide on dentin matrix and resin-dentin bonds. J. Biomed. Mater. Res. B Appl. Biomater..

[27] Vidal C.M., Zhu W., Manohar S., Aydin B., Keiderling T.A., Messersmith P., Bedran-Russo A.K. (2016). Collagen-collagen interactions mediated by plant-derived proanthocyanidins: A spectroscopic and atomic force microscopy study. Acta Biomater..

[28] Olde Damink L.H., Dijkstra P.J., van Luyn M.J., van Wachem P.B., Nieuwenhuis P., Feijen J. (1996). Cross-linking of dermal sheep collagen using a water-soluble carbodiimide. Biomaterials.

[29] Macedo G., Yamauchi M., Bedran-Russo A. (2009). Effects of Chemical Cross-linkers on Caries-affected Dentin Bonding. J. Dent. Res..

[30] Timkovich R. (1977). Detection of the stable addition of carbodiimide to proteins. Anal. Biochem..

[31] Staros J.V., Wright R.W., Swingle D.M. (1986). Enhancement by N-hydroxysulfosuccinimide of water-soluble carbodiimide-mediated coupling reactions. Anal. Biochem..

[32] Mazzoni A., Apolonio F.M., Saboia V.P.A., Santi S., Angeloni V., Checchi V., Curci R., Di Lenarda R., Tay F., Pashley D.H. (2014). Carbodiimide Inactivation of MMPs and Effect on Dentin Bonding. J. Dent. Res..

[33] Mazzoni A., Nascimento F., Carrilho M., Tersariol I., Papa V., Tjäderhane L., Di Lenarda R., Tay F., Pashley D., Breschi L. (2012). MMP Activity in the Hybrid Layer Detected with in situ Zymography. J. Dent. Res..

[34] Ku C., Sathishkumar M., Mun S. (2007). Binding affinity of proanthocyanidin from waste Pinus radiata bark onto proline-rich bovine achilles tendon collagen type I. Chemosphere.

[35] Haslam E., Lilley T.H. (1986). Interactions of natural phenols with macromolecules. Prog. Clin. Boil. Res..

[36] Miles C.A., Avery N.C., Rodin V.V., Bailey A.J. (2005). The Increase in Denaturation Temperature Following Cross-linking of Collagen is Caused by Dehydration of the Fibres. J. Mol. Biol..

[37] He L., Mu C., Shi J., Zhang Q., Shi B., Lin W. (2011). Modification of collagen with a natural cross-linker, procyanidin. Int. J. Biol. Macromol..

[38] Bedran-Russo A.K.B., Pashley D.H., Agee K., Drummond J.L., Miescke K.J. (2008). Changes in stiffness of demineralized dentin following application of collagen crosslinkers. J. Biomed. Mater. Res. Part B Appl. Biomater..

[39] Oliveira P., Montembault A., Sudre G., Alcouffe P., Marcon L., Gehan H., Lux F., Albespy K., Centis V., Campos D. (2019). Self-crosslinked fibrous collagen/chitosan blends: Processing, properties evaluation and monitoring of degradation by bi-fluorescence imaging. Int. J. Biol. Macromol..

[40] Júnior Z.S.S., Botta S.B., Ana P.A., Franca C., Fernandes K., Mesquita-Ferrari R.A., Deana A., Bussadori S.K. (2015). Effect of papain-based gel on type I collagen-spectroscopy applied for microstructural analysis. Sci. Rep..

[41] Nam K., Kimura T., Kishida A. (2008). Controlling coupling reaction of EDC and NHS for preparation of collagen gels using etha-nol/water co-solvents. Macromol. Biosci..

[42] Madhan B., Krishnamoorthy G., Rao J.R., Nair B.U. (2007). Role of green tea polyphenols in the inhibition of collagenolytic activity by collagenase. Int. J. Biol. Macromol..

[43] Pal G.K., Suresh P.V. (2016). Microbial collagenases: Challenges and prospects in production and potential applications in food and nutrition. RSC Adv..

[44] Moreira M., Souza N., Sousa R., Freitas D., Lemos M., De Paula D., Maia F., Lomonaco D., Mazzetto S., Feitosa V. (2017). Efficacy of new natural biomodification agents from Anacardiaceae extracts on dentin collagen cross-linking. Dent. Mater..

[45] Aydin B., Leme-Kraus A.A., Vidal C.M., Aguiar T.R., Phansalkar R.S., Nam J.-W., McAlpine J.B., Chen S.-N., Pauli G.F., Bedran-Russo A.K. (2019). Evidence to the role of interflavan linkages and galloylation of proanthocyanidins at sustaining long-term dentin biomodification. Dent. Mater..

[46] Xu Z., Wei L.-H., Ge Z.-Z., Zhu W., Li C.-M. (2014). Comparison of the degradation kinetics of A-type and B-type proanthocyanidins dimers as a function of pH and temperature. Eur. Food Res. Technol..

[47] Hermanson G.T., Hermanson G.T. (2013). Chapter 4—Zero-Length Crosslinkers. Bioconjugate Techniques.

